# Pd_2.28(1)_Zn_10.37(1)_Al_0.35(1)_, a ternary γ-brass-type structure

**DOI:** 10.1107/S1600536810000723

**Published:** 2010-01-13

**Authors:** Srinivasa Thimmaiah, Gordon J. Miller

**Affiliations:** a Department of Chemistry and Ames Laboratory, Iowa State University, Ames, IA 50011, USA

## Abstract

Palladium zinc aluminium (2.28/10.37/0.35), Pd_2.28(1)_Zn_10.37(1)_Al_0.35(1)_, represents the upper limit of Al substitution into the parent cubic γ-brass Pd_2+*x*_Zn_11−*x*_. The structure can be described in terms of a 26-atom cluster consisting of an inner tetra­hedron (IT), an outer tetra­hedron (OT), an octa­hedron (OH) and a cubocta­hedron (CO), with the substituted Al atoms partially occupying the IT (.3*m*) and CO (..*m*) sites.

## Related literature

For related literature, see: Arnberg & Westman (1972 ▶); Edström & Westman (1969 ▶); Gross *et al.* (2001 ▶); Gourdon & Miller (2006 ▶); Harbrecht *et al.* (2002 ▶); Thimmaiah & Miller (2010 ▶). For standardization of crystal structures, see: Gelato & Parthé (1987 ▶).

## Experimental

### 

#### Crystal data

Pd_2.28_Zn_10.37_Al_0.35_
*M* *_r_* = 929.56
Cubic, 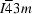

*a* = 9.1079 (11) Å
*V* = 755.54 (16) Å^3^
*Z* = 4
Mo *K*α radiation
μ = 37.46 mm^−1^
*T* = 293 K
0.12 × 0.06 × 0.03 mm

#### Data collection

Stoe IPDS-II diffractometer
Absorption correction: numerical (*X-SHAPE* and *X-RED*; Stoe & Cie, 2005 ▶) *T*_min_ = 0.054, *T*_max_ = 0.465
11243 measured reflections
339 independent reflections
337 reflections with *I* > 2σ(*I*)
*R*_int_ = 0.069

#### Refinement

*R*[*F* ^2^ > 2σ(*F* ^2^)] = 0.027
*wR*(*F* ^2^) = 0.068
*S* = 1.02
339 reflections
22 parameters
Δρ_max_ = 1.05 e Å^−3^
Δρ_min_ = −1.19 e Å^−3^
Absolute structure: Flack (1983 ▶)
Flack parameter: 0.04 (4)

### 

Data collection: *X-AREA* (Stoe & Cie, 2009 ▶); cell refinement: *X-AREA*; data reduction: *X-AREA*; program(s) used to solve structure: *SHELXS97* (Sheldrick, 2008 ▶); program(s) used to refine structure: *SHELXL97* (Sheldrick, 2008 ▶); molecular graphics: *DIAMOND* (Brandenburg, 2009 ▶); software used to prepare material for publication: *SHELXTL* (Sheldrick, 2008 ▶).

## Supplementary Material

Crystal structure: contains datablocks I, global. DOI: 10.1107/S1600536810000723/mg2090sup1.cif

Structure factors: contains datablocks I. DOI: 10.1107/S1600536810000723/mg2090Isup2.hkl

Additional supplementary materials:  crystallographic information; 3D view; checkCIF report

## Figures and Tables

**Fig. 1.**
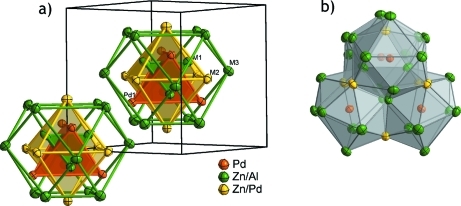
Cubic γ-brass structure of Pd2.28 (1)Zn10.37 (1)Al0.35 (1) in terms of (a) 26-atom clusters in bcc arrangement, with different polyhedra emphasized, and (b) four interpenetrating Pd-centered icosahedra. The color scheme is shown and displacement ellipsoids are drawn at the 90% probability level.

## supplementary crystallographic information

### Comment

Various *M*_2_Zn_11_ phases [*M* = Rh (Gross *et al.*, 2001), Pd (Gourdon & Miller, 2006), Ir (Arnberg & Westman, 1972), Pt (Harbrecht *et al.*, 2002)] adopt the γ-brass type structure (Pearson code *cI*52). To study the influence of valence electron concentration (vec) on γ-brass type phases, we attempted replacing Zn by Al in the parent Pd_2 + _*_x_*Zn_11 - _*_x_* phase (Gourdon & Miller, 2006). Initially obtained as a side product, Pd_2.28 (1)_Zn_10.37 (1)_Al_0.35 (1)_ represents the upper limit of Al substitution in the Pd_2 + _*_x_*Zn_11 - _*_x_* phase. Further substitution of Al leads to 2×2×2 superstructures of γ-brass with lattice parameters ranging from 18.0700 (3) to 18.1600 (2) Å (Pearson code *cF*400–*cF*416) (Thimmaiah & Miller, 2010).

In terms of the 26-atom clusters (in *bcc* arrangement) commonly used to describe the structure of γ-brass, the inner tetrahedron (IT) and cuboctahedron (CO) are occupied by mixtures of Zn and Al atoms, the outer tetrahedron (OT) is fully occupied by Pd atoms, and the octahedron (OH) is occupied by a mixture of Zn and Pd atoms (Fig. 1a). Similar mixing of Zn and Pd atoms on the OH sites is observed in binary Pd_2 + _*_x_*Zn_11 - _*_x_* (Gourdon & Miller, 2006; Edström & Westman, 1969). An alternative description involves four interpenetrating icosahedra, which are constructed around each OT atom and encapsulate a tetrahedron formed by IT atoms (Fig. 1 b). The IT and OT sites are each surrounded by 12 nearest neighbours [at distances of 2.666 (1)–2.789 (2) Å and 2.624 (1)–2.794 (1) Å, respectively] forming distorted icosahedra. On the other hand, the coordination numbers are 13 around the OH site [2.591 (2)–2.945 (1) Å] and 11 around the CO site [2.612 (1)–2.945 (1) Å].

### Experimental

The title compound was prepared from 0.5 - g mixtures of the elements (Pd foil, MPC, Ames Laboratory, 99.999%; Zn ingot, MPC, Ames Laboratory, 99.999%; Al tear drop, MPC, Ames Laboratory, 99.999%) loaded into cleaned Ta tubes, which were placed in evacuated (10^-5^ torr) and sealed silica tubes. The tubes were heated at 30 °C h^-1^ to 850 °C, kept there for 12 h, cooled to 550 °C over 12 h, equilibrated there for 3 d, and then cooled to room temperature by shutting off the furnace.

### Refinement

Refinement of a starting model (Gourdon & Miller, 2006) led to a mixture of 0.09 (3) Pd and 0.91 (3) Zn in the OH sites. However, the IT and CO sites, initially assumed to be fully occupied by Zn atoms, exhibited elevated isotropic displacement parameters. Modeling these sites with a mixture of Zn and Al resulted in the refined composition Pd_2.28 (1)_Zn_10.37 (1)_Al_0.35 (1)_. Analysis of multiple crystals obtained from the same and other batches gave the same site occupancies. Within the limitation of the technique, semiquantitative energy-dispersive X-ray analysis corroborate this chemical composition. The structure was standardized by means of the program *STRUCTURE TIDY* (Gelato & Parthé, 1987). The highest peak and the deepest hole are located 1.26 Å and 1.18 Å, respectively, from Pd1.

### Crystal data

**Table:**

Pd_2.28_Zn_10.37_Al_0.35_	Melting point: not measured K
*M**_r_* = 929.56	Mo *K*α radiation, λ = 0.71073 Å
Cubic, *I*43*m*	Cell parameters from 2000 reflections
Hall symbol: I -4 2 3	θ = 3.2–34.8°
*a* = 9.1079 (11) Å	µ = 37.46 mm^−^^1^
*V* = 755.54 (16) Å^3^	*T* = 293 K
*Z* = 4	Rectangular, silver
*F*(000) = 1681	0.12 × 0.06 × 0.03 mm
*D*_x_ = 8.172 Mg m^−^^3^	

### Data collection

**Table:**

Stoe/IPDS-II diffractometer	339 independent reflections
Radiation source: fine-focus sealed tube	337 reflections with *I* > 2σ(*I*)
graphite	*R*_int_ = 0.069
φ and ω scans	θ_max_ = 34.8°, θ_min_ = 3.2°
Absorption correction: numerical (*X-SHAPE* and *X-RED*; Stoe & Cie, 2005)	*h* = −14→14
*T*_min_ = 0.054, *T*_max_ = 0.465	*k* = −14→13
11243 measured reflections	*l* = −14→14

### Refinement

**Table:**

Refinement on *F*^2^	Secondary atom site location: difference Fourier map
Least-squares matrix: full	*w* = 1/[σ^2^(*F*_o_^2^) + (0.0249*P*)^2^ + 32.9721*P*] where *P* = (*F*_o_^2^ + 2*F*_c_^2^)/3
*R*[*F*^2^ > 2σ(*F*^2^)] = 0.027	(Δ/σ)_max_ < 0.001
*wR*(*F*^2^) = 0.068	Δρ_max_ = 1.05 e Å^−^^3^
*S* = 1.02	Δρ_min_ = −1.19 e Å^−^^3^
339 reflections	Extinction correction: *SHELXL97* (Sheldrick, 2008), Fc^*^=kFc[1+0.001xFc^2^λ^3^/sin(2θ)]^-1/4^
22 parameters	Extinction coefficient: 0.00118 (16)
0 restraints	Absolute structure: Flack (1983)
Primary atom site location: structure-invariant direct methods	Flack parameter: 0.04 (4)

### Special details

**Table:**

Geometry. All e.s.d.'s (except the e.s.d. in the dihedral angle between two l.s. planes) are estimated using the full covariance matrix. The cell e.s.d.'s are taken into account individually in the estimation of e.s.d.'s in distances, angles and torsion angles; correlations between e.s.d.'s in cell parameters are only used when they are defined by crystal symmetry. An approximate (isotropic) treatment of cell e.s.d.'s is used for estimating e.s.d.'s involving l.s. planes.
Refinement. Refinement of *F*^2^ against ALL reflections. The weighted *R*-factor *wR* and goodness of fit *S* are based on *F*^2^, conventional *R*-factors *R* are based on *F*, with *F* set to zero for negative *F*^2^. The threshold expression of *F*^2^ > σ(*F*^2^) is used only for calculating *R*-factors(gt) *etc*. and is not relevant to the choice of reflections for refinement. *R*-factors based on *F*^2^ are statistically about twice as large as those based on *F*, and *R*- factors based on ALL data will be even larger.

### Fractional atomic coordinates and isotropic or equivalent isotropic displacement parameters (Å^2^)

**Table:**

	*x*	*y*	*z*	*U*_iso_*/*U*_eq_	Occ. (<1)
Pd1	0.32674 (6)	0.32674 (6)	0.32674 (6)	0.0101 (3)	
Zn1	0.10828 (12)	0.10828 (12)	0.10828 (12)	0.0139 (5)	0.924 (17)
Al1	0.10828 (12)	0.10828 (12)	0.10828 (12)	0.0139 (5)	0.076 (17)
Zn2	0.35776 (15)	0.0000	0.0000	0.0135 (5)	0.91 (3)
Pd2	0.35776 (15)	0.0000	0.0000	0.0135 (5)	0.09 (3)
Zn3	0.31076 (9)	0.31076 (9)	0.03932 (12)	0.0162 (3)	0.966 (13)
Al3	0.31076 (9)	0.31076 (9)	0.03932 (12)	0.0162 (3)	0.034 (13)

### Atomic displacement parameters (Å^2^)

**Table:**

	*U*^11^	*U*^22^	*U*^33^	*U*^12^	*U*^13^	*U*^23^
Pd1	0.0101 (3)	0.0101 (3)	0.0101 (3)	0.0007 (2)	0.0007 (2)	0.0007 (2)
Zn1	0.0139 (5)	0.0139 (5)	0.0139 (5)	0.0029 (4)	0.0029 (4)	0.0029 (4)
Al1	0.0139 (5)	0.0139 (5)	0.0139 (5)	0.0029 (4)	0.0029 (4)	0.0029 (4)
Zn2	0.0124 (7)	0.0140 (6)	0.0140 (6)	0.000	0.000	0.0034 (5)
Pd2	0.0124 (7)	0.0140 (6)	0.0140 (6)	0.000	0.000	0.0034 (5)
Zn3	0.0177 (4)	0.0177 (4)	0.0132 (5)	−0.0026 (3)	−0.0028 (2)	−0.0028 (2)
Al3	0.0177 (4)	0.0177 (4)	0.0132 (5)	−0.0026 (3)	−0.0028 (2)	−0.0028 (2)

### Geometric parameters (Å, °)

**Table:**

Pd1—Al3^i^	2.6240 (11)	Zn1—Zn3	2.6826 (17)
Pd1—Zn3^i^	2.6240 (11)	Zn2—Pd2^vi^	2.591 (3)
Pd1—Al3^ii^	2.6240 (11)	Zn2—Zn2^vi^	2.591 (3)
Pd1—Al3^iii^	2.6240 (11)	Zn2—Al3^vii^	2.6115 (12)
Pd1—Zn3^ii^	2.6240 (11)	Zn2—Zn3^vii^	2.6115 (12)
Pd1—Zn3^iii^	2.6240 (11)	Zn2—Al3^viii^	2.6115 (12)
Pd1—Al3^iv^	2.6259 (12)	Zn2—Zn3^viii^	2.6115 (12)
Pd1—Zn3^iv^	2.6259 (12)	Zn2—Zn1^ix^	2.6662 (12)
Pd1—Al3^v^	2.6259 (12)	Zn2—Al1^ix^	2.6662 (12)
Pd1—Zn3^v^	2.6259 (12)	Zn2—Pd1^x^	2.7936 (9)
Pd1—Zn3	2.6259 (12)	Zn2—Pd1^xi^	2.7936 (9)
Zn1—Zn2	2.6662 (12)	Zn3—Pd2^i^	2.6115 (12)
Zn1—Pd2^v^	2.6662 (12)	Zn3—Zn2^i^	2.6115 (12)
Zn1—Pd2^iv^	2.6662 (12)	Zn3—Pd1^x^	2.6240 (11)
Zn1—Zn2^v^	2.6662 (12)	Zn3—Al3^xii^	2.7245 (6)
Zn1—Zn2^iv^	2.6662 (12)	Zn3—Al3^vii^	2.7245 (6)
Zn1—Al3^v^	2.6826 (17)	Zn3—Zn3^xii^	2.7245 (6)
Zn1—Al3^iv^	2.6826 (17)	Zn3—Zn3^vii^	2.7245 (6)
Zn1—Zn3^v^	2.6826 (17)	Zn3—Al3^i^	2.7245 (6)
Zn1—Zn3^iv^	2.6826 (17)	Zn3—Al3^ii^	2.7245 (6)
			
Al3^i^—Pd1—Zn3^i^	0.00 (6)	Al3^vii^—Zn2—Zn3^vii^	0.00 (5)
Al3^i^—Pd1—Al3^ii^	118.459 (14)	Pd2^vi^—Zn2—Al3^viii^	68.97 (4)
Zn3^i^—Pd1—Al3^ii^	118.459 (14)	Zn2^vi^—Zn2—Al3^viii^	68.97 (4)
Al3^i^—Pd1—Al3^iii^	118.459 (14)	Al3^vii^—Zn2—Al3^viii^	137.93 (7)
Zn3^i^—Pd1—Al3^iii^	118.459 (14)	Zn3^vii^—Zn2—Al3^viii^	137.93 (7)
Al3^ii^—Pd1—Al3^iii^	118.459 (14)	Pd2^vi^—Zn2—Zn3^viii^	68.97 (4)
Al3^i^—Pd1—Zn3^ii^	118.459 (14)	Zn2^vi^—Zn2—Zn3^viii^	68.97 (4)
Zn3^i^—Pd1—Zn3^ii^	118.459 (14)	Al3^vii^—Zn2—Zn3^viii^	137.93 (7)
Al3^ii^—Pd1—Zn3^ii^	0.00 (6)	Zn3^vii^—Zn2—Zn3^viii^	137.93 (7)
Al3^iii^—Pd1—Zn3^ii^	118.459 (14)	Al3^viii^—Zn2—Zn3^viii^	0.00 (3)
Al3^i^—Pd1—Zn3^iii^	118.459 (14)	Pd2^vi^—Zn2—Zn1	148.46 (4)
Zn3^i^—Pd1—Zn3^iii^	118.459 (14)	Zn2^vi^—Zn2—Zn1	148.46 (4)
Al3^ii^—Pd1—Zn3^iii^	118.459 (14)	Al3^vii^—Zn2—Zn1	107.81 (3)
Al3^iii^—Pd1—Zn3^iii^	0.00 (6)	Zn3^vii^—Zn2—Zn1	107.81 (3)
Zn3^ii^—Pd1—Zn3^iii^	118.459 (14)	Al3^viii^—Zn2—Zn1	107.81 (3)
Al3^i^—Pd1—Al3^iv^	62.53 (3)	Zn3^viii^—Zn2—Zn1	107.81 (3)
Zn3^i^—Pd1—Al3^iv^	62.53 (3)	Pd2^vi^—Zn2—Zn1^ix^	148.46 (4)
Al3^ii^—Pd1—Al3^iv^	133.05 (4)	Zn2^vi^—Zn2—Zn1^ix^	148.46 (4)
Al3^iii^—Pd1—Al3^iv^	62.53 (3)	Al3^vii^—Zn2—Zn1^ix^	107.81 (3)
Zn3^ii^—Pd1—Al3^iv^	133.05 (4)	Zn3^vii^—Zn2—Zn1^ix^	107.81 (3)
Zn3^iii^—Pd1—Al3^iv^	62.53 (3)	Al3^viii^—Zn2—Zn1^ix^	107.81 (3)
Al3^i^—Pd1—Zn3^iv^	62.53 (3)	Zn3^viii^—Zn2—Zn1^ix^	107.81 (3)
Zn3^i^—Pd1—Zn3^iv^	62.53 (3)	Zn1—Zn2—Zn1^ix^	63.08 (9)
Al3^ii^—Pd1—Zn3^iv^	133.05 (4)	Pd2^vi^—Zn2—Al1^ix^	148.46 (4)
Al3^iii^—Pd1—Zn3^iv^	62.53 (3)	Zn2^vi^—Zn2—Al1^ix^	148.46 (4)
Zn3^ii^—Pd1—Zn3^iv^	133.05 (4)	Al3^vii^—Zn2—Al1^ix^	107.81 (3)
Zn3^iii^—Pd1—Zn3^iv^	62.53 (3)	Zn3^vii^—Zn2—Al1^ix^	107.81 (3)
Al3^iv^—Pd1—Zn3^iv^	0.00 (7)	Al3^viii^—Zn2—Al1^ix^	107.81 (3)
Al3^i^—Pd1—Al3^v^	133.05 (4)	Zn3^viii^—Zn2—Al1^ix^	107.81 (3)
Zn3^i^—Pd1—Al3^v^	133.05 (4)	Zn1—Zn2—Al1^ix^	63.08 (9)
Al3^ii^—Pd1—Al3^v^	62.53 (3)	Zn1^ix^—Zn2—Al1^ix^	0.00 (6)
Al3^iii^—Pd1—Al3^v^	62.53 (3)	Pd2^vi^—Zn2—Pd1^x^	126.98 (3)
Zn3^ii^—Pd1—Al3^v^	62.53 (3)	Zn2^vi^—Zn2—Pd1^x^	126.98 (3)
Zn3^iii^—Pd1—Al3^v^	62.53 (3)	Al3^vii^—Zn2—Pd1^x^	58.01 (3)
Al3^iv^—Pd1—Al3^v^	83.48 (4)	Zn3^vii^—Zn2—Pd1^x^	58.01 (3)
Zn3^iv^—Pd1—Al3^v^	83.48 (4)	Al3^viii^—Zn2—Pd1^x^	164.05 (6)
Al3^i^—Pd1—Zn3^v^	133.05 (4)	Zn3^viii^—Zn2—Pd1^x^	164.05 (6)
Zn3^i^—Pd1—Zn3^v^	133.05 (4)	Zn1—Zn2—Pd1^x^	59.16 (3)
Al3^ii^—Pd1—Zn3^v^	62.53 (3)	Zn1^ix^—Zn2—Pd1^x^	59.16 (3)
Al3^iii^—Pd1—Zn3^v^	62.53 (3)	Al1^ix^—Zn2—Pd1^x^	59.16 (3)
Zn3^ii^—Pd1—Zn3^v^	62.53 (3)	Pd2^vi^—Zn2—Pd1^xi^	126.98 (3)
Zn3^iii^—Pd1—Zn3^v^	62.53 (3)	Zn2^vi^—Zn2—Pd1^xi^	126.98 (3)
Al3^iv^—Pd1—Zn3^v^	83.48 (4)	Al3^vii^—Zn2—Pd1^xi^	164.05 (6)
Zn3^iv^—Pd1—Zn3^v^	83.48 (4)	Zn3^vii^—Zn2—Pd1^xi^	164.05 (6)
Al3^v^—Pd1—Zn3^v^	0.00 (7)	Al3^viii^—Zn2—Pd1^xi^	58.01 (3)
Al3^i^—Pd1—Zn3	62.53 (3)	Zn3^viii^—Zn2—Pd1^xi^	58.01 (3)
Zn3^i^—Pd1—Zn3	62.53 (3)	Zn1—Zn2—Pd1^xi^	59.16 (3)
Al3^ii^—Pd1—Zn3	62.53 (3)	Zn1^ix^—Zn2—Pd1^xi^	59.16 (3)
Al3^iii^—Pd1—Zn3	133.05 (4)	Al1^ix^—Zn2—Pd1^xi^	59.16 (3)
Zn3^ii^—Pd1—Zn3	62.53 (3)	Pd1^x^—Zn2—Pd1^xi^	106.04 (6)
Zn3^iii^—Pd1—Zn3	133.05 (4)	Pd2^i^—Zn3—Zn2^i^	0.00 (5)
Al3^iv^—Pd1—Zn3	83.48 (4)	Pd2^i^—Zn3—Pd1^x^	153.49 (6)
Zn3^iv^—Pd1—Zn3	83.48 (4)	Zn2^i^—Zn3—Pd1^x^	153.49 (6)
Al3^v^—Pd1—Zn3	83.48 (4)	Pd2^i^—Zn3—Pd1	64.47 (4)
Zn3^v^—Pd1—Zn3	83.48 (4)	Zn2^i^—Zn3—Pd1	64.47 (4)
Zn2—Zn1—Pd2^v^	119.582 (10)	Pd1^x^—Zn3—Pd1	142.04 (5)
Zn2—Zn1—Pd2^iv^	119.582 (10)	Pd2^i^—Zn3—Zn1	145.42 (6)
Pd2^v^—Zn1—Pd2^iv^	119.582 (10)	Zn2^i^—Zn3—Zn1	145.42 (6)
Zn2—Zn1—Zn2^v^	119.582 (10)	Pd1^x^—Zn3—Zn1	61.09 (5)
Pd2^v^—Zn1—Zn2^v^	0.0	Pd1—Zn3—Zn1	80.96 (5)
Pd2^iv^—Zn1—Zn2^v^	119.582 (10)	Pd2^i^—Zn3—Al3^xii^	102.22 (4)
Zn2—Zn1—Zn2^iv^	119.582 (10)	Zn2^i^—Zn3—Al3^xii^	102.22 (4)
Pd2^v^—Zn1—Zn2^iv^	119.582 (10)	Pd1^x^—Zn3—Al3^xii^	58.77 (4)
Pd2^iv^—Zn1—Zn2^iv^	0.0	Pd1—Zn3—Al3^xii^	139.15 (3)
Zn2^v^—Zn1—Zn2^iv^	119.582 (10)	Zn1—Zn3—Al3^xii^	104.13 (5)
Zn2—Zn1—Al3^v^	65.28 (2)	Pd2^i^—Zn3—Al3^vii^	102.22 (4)
Pd2^v^—Zn1—Al3^v^	65.28 (2)	Zn2^i^—Zn3—Al3^vii^	102.22 (4)
Pd2^iv^—Zn1—Al3^v^	135.08 (8)	Pd1^x^—Zn3—Al3^vii^	58.77 (4)
Zn2^v^—Zn1—Al3^v^	65.28 (2)	Pd1—Zn3—Al3^vii^	139.15 (3)
Zn2^iv^—Zn1—Al3^v^	135.08 (8)	Zn1—Zn3—Al3^vii^	104.13 (5)
Zn2—Zn1—Al3^iv^	135.08 (8)	Al3^xii^—Zn3—Al3^vii^	79.83 (8)
Pd2^v^—Zn1—Al3^iv^	65.28 (2)	Pd2^i^—Zn3—Zn3^xii^	102.22 (4)
Pd2^iv^—Zn1—Al3^iv^	65.28 (2)	Zn2^i^—Zn3—Zn3^xii^	102.22 (4)
Zn2^v^—Zn1—Al3^iv^	65.28 (2)	Pd1^x^—Zn3—Zn3^xii^	58.77 (4)
Zn2^iv^—Zn1—Al3^iv^	65.28 (2)	Pd1—Zn3—Zn3^xii^	139.15 (3)
Al3^v^—Zn1—Al3^iv^	81.33 (6)	Zn1—Zn3—Zn3^xii^	104.13 (5)
Zn2—Zn1—Zn3^v^	65.28 (2)	Al3^xii^—Zn3—Zn3^xii^	0.00 (6)
Pd2^v^—Zn1—Zn3^v^	65.28 (2)	Al3^vii^—Zn3—Zn3^xii^	79.83 (8)
Pd2^iv^—Zn1—Zn3^v^	135.08 (8)	Pd2^i^—Zn3—Zn3^vii^	102.22 (4)
Zn2^v^—Zn1—Zn3^v^	65.28 (2)	Zn2^i^—Zn3—Zn3^vii^	102.22 (4)
Zn2^iv^—Zn1—Zn3^v^	135.08 (8)	Pd1^x^—Zn3—Zn3^vii^	58.77 (4)
Al3^v^—Zn1—Zn3^v^	0.00 (6)	Pd1—Zn3—Zn3^vii^	139.15 (3)
Al3^iv^—Zn1—Zn3^v^	81.33 (6)	Zn1—Zn3—Zn3^vii^	104.13 (5)
Zn2—Zn1—Zn3^iv^	135.08 (8)	Al3^xii^—Zn3—Zn3^vii^	79.83 (8)
Pd2^v^—Zn1—Zn3^iv^	65.28 (2)	Al3^vii^—Zn3—Zn3^vii^	0.00 (4)
Pd2^iv^—Zn1—Zn3^iv^	65.28 (2)	Zn3^xii^—Zn3—Zn3^vii^	79.83 (8)
Zn2^v^—Zn1—Zn3^iv^	65.28 (2)	Pd2^i^—Zn3—Al3^i^	65.41 (4)
Zn2^iv^—Zn1—Zn3^iv^	65.28 (2)	Zn2^i^—Zn3—Al3^i^	65.41 (4)
Al3^v^—Zn1—Zn3^iv^	81.33 (6)	Pd1^x^—Zn3—Al3^i^	122.72 (4)
Al3^iv^—Zn1—Zn3^iv^	0.00 (6)	Pd1—Zn3—Al3^i^	58.70 (3)
Zn3^v^—Zn1—Zn3^iv^	81.33 (6)	Zn1—Zn3—Al3^i^	97.39 (5)
Zn2—Zn1—Zn3	65.28 (2)	Al3^xii^—Zn3—Al3^i^	80.50 (3)
Pd2^v^—Zn1—Zn3	135.08 (8)	Al3^vii^—Zn3—Al3^i^	153.76 (7)
Pd2^iv^—Zn1—Zn3	65.28 (2)	Zn3^xii^—Zn3—Al3^i^	80.50 (3)
Zn2^v^—Zn1—Zn3	135.08 (8)	Zn3^vii^—Zn3—Al3^i^	153.76 (7)
Zn2^iv^—Zn1—Zn3	65.28 (2)	Pd2^i^—Zn3—Al3^ii^	65.41 (4)
Al3^v^—Zn1—Zn3	81.33 (6)	Zn2^i^—Zn3—Al3^ii^	65.41 (4)
Al3^iv^—Zn1—Zn3	81.33 (6)	Pd1^x^—Zn3—Al3^ii^	122.72 (4)
Zn3^v^—Zn1—Zn3	81.33 (6)	Pd1—Zn3—Al3^ii^	58.70 (3)
Zn3^iv^—Zn1—Zn3	81.33 (6)	Zn1—Zn3—Al3^ii^	97.39 (5)
Pd2^vi^—Zn2—Zn2^vi^	0.0	Al3^xii^—Zn3—Al3^ii^	153.76 (7)
Pd2^vi^—Zn2—Al3^vii^	68.97 (4)	Al3^vii^—Zn3—Al3^ii^	80.50 (3)
Zn2^vi^—Zn2—Al3^vii^	68.97 (4)	Zn3^xii^—Zn3—Al3^ii^	153.76 (7)
Pd2^vi^—Zn2—Zn3^vii^	68.97 (4)	Zn3^vii^—Zn3—Al3^ii^	80.50 (3)
Zn2^vi^—Zn2—Zn3^vii^	68.97 (4)	Al3^i^—Zn3—Al3^ii^	111.69 (7)

Symmetry codes: (i) −*y*+1/2, *z*+1/2, −*x*+1/2; (ii) *z*+1/2, −*x*+1/2, −*y*+1/2; (iii) −*x*+1/2, −*y*+1/2, *z*+1/2; (iv) *z*, *x*, *y*; (v) *y*, *z*, *x*; (vi) −*x*+1, −*y*, *z*; (vii) −*z*+1/2, −*x*+1/2, *y*−1/2; (viii) −*z*+1/2, *x*−1/2, −*y*+1/2; (ix) *x*, −*y*, −*z*; (x) −*x*+1/2, −*y*+1/2, *z*−1/2; (xi) −*x*+1/2, *y*−1/2, −*z*+1/2; (xii) −*y*+1/2, −*z*+1/2, *x*−1/2.
